# Gaps in antihypertensive and statin treatments and benefits of optimisation: a modelling study in a 1 million ethnically diverse urban population in UK

**DOI:** 10.1136/bmjopen-2021-052884

**Published:** 2021-12-30

**Authors:** Runguo Wu, Stuart Christopher Gorthorn Rison, Zahra Raisi-Estabragh, Isabel Dostal, Chris Carvalho, John Robson, Borislava Mihaylova

**Affiliations:** 1 Wolfson Institute of Population Health, Barts and the London School of Medicine and Dentistry, Queen Mary University of London, London, UK; 2 Bromley-by-Bow Health Centre, London, UK; 3 William Harvey Research Institute, NIHR Barts Biomedical Research Centre, Queen Mary University of London, London, UK; 4 Barts Heart Centre, St Bartholomew’s Hospital, Barts Health NHS Trust, London, UK; 5 De Beauvoir Surgery, London, UK; 6 Nuffield Department of Population Health, University of Oxford, Oxford, UK

**Keywords:** hypertension, primary care, preventive medicine, quality in health care, health policy, ischaemic heart disease

## Abstract

**Objectives:**

To characterise gaps in antihypertensive treatment in people with hypertension and statin treatment in people with cardiovascular diseases (CVD) in a large urban population and quantify the health and economic impacts of their optimisation.

**Design:**

A cross-sectional population study and a long-term CVD decision model.

**Setting:**

Primary care, UK.

**Participants:**

All adults with diagnosed hypertension or CVD in a population of about 1 million people, served by 123 primary care practices in London, UK in 2019.

**Interventions:**

Following UK clinical guidelines, all adults with diagnosed hypertension were categorised into optimal, suboptimal and untreated groups with respect to their antihypertensive treatment, and all adults with diagnosed CVD were categorised in the same manner with respect to their statin treatment.

**Outcomes:**

Proportion of patients suboptimally treated or untreated. Projected cardiovascular events avoided, years and quality-adjusted life years (QALYs) gained and healthcare costs saved with optimised treatments.

**Results:**

21 954 of the 91 828 adults with hypertension (24%; mean age 59 years; 49% women) and 9062 of the 23 723 adults with CVD (38%; mean age 69 years; 43% women) were not optimally treated with antihypertensive or statin treatment, respectively. Per 1000 additional patients optimised over 5 years, hypertension treatment is projected to prevent 25 (95% CI 16 to 32) major vascular events (MVEs) and 7 (3 to 10) vascular deaths, statin treatment, 28 (22 to 33) MVEs and 6 (4 to 7) vascular deaths. Over their lifespan, a patient with uncontrolled hypertension aged 60–69 years is projected to gain 0.64 (95% CI 0.36 to 0.87) QALYs with optimised hypertension treatment, and a similarly aged patient with previous CVD not optimally treated with statin is projected to gain 0.3 (0.24 to 0.37) QALYs with optimised statin treatment. In both cases, the hospital cost savings minus extra medication costs were about £1100 per person over remaining lifespan.

**Conclusions:**

Optimising cardiovascular treatments can cost-effectively reduce cardiovascular risk and improve life expectancy.

**Strengths and limitations of this study**

- This study quantifies the gaps in blood pressure and statin treatments among people at high cardiovascular disease risk in a large ethnically diverse UK urban population.
- A cardiovascular disease model projects net health outcomes and extra healthcare costs with treatment optimisation to guide prioritisation of efforts.
- The study does not assess specific interventions to improve uptake and adherence to recommended treatments.

## Introduction

Cardiovascular disease (CVD) is the most common cause of morbidity and mortality worldwide, and hypertension and hypercholesterolaemia are two of its key modifiable risk factors.^1^ Their widespread suboptimal treatment, however, represents a substantial missed opportunity for CVD prevention.^2^ Reducing raised systolic blood pressure by 10 mm Hg with antihypertensive treatment decreases risks of ischaemic heart disease (IHD) and stroke by 25%–35%,^3 4^ and reducing low-density lipoprotein cholesterol (LDL-C) by 1 mmol/L with statin therapy reduces these risks by 24%–25% with more intensive statin regimens achieving larger risk reductions.^5^ In the UK, the National Institute for Health and Care Excellence (NICE) recommends antihypertensive medications for people with high blood pressure^6^ and high-intensity statin treatment for people with CVD.^7^ However, gaps in treatment initiation and poor patient adherence to treatment are common^8 9^ and a strategy to highlight the benefits and complexity of treating to targets, and to structure routine practice to facilitate medicines optimisation, has been put forward.^10^

East London is a geographic area in London, UK with an ethnically diverse urban population. More than 70% of east London areas are categorised in the bottom two quintiles of country’s socioeconomic deprivation.^11^ Primary care practices in east London are looking to implement a programme to optimise blood pressure and lipid control to improve quality of CVD management and reduce health inequality in the population. In this study, we describe the gaps in antihypertensive and statin treatments in east London and project the health and healthcare costs with their optimisation.

## Methods

### Study population

Data were extracted for all adult patients (aged ≥18 years on 1 January 2020) with diagnosis of hypertension or CVD among a population of about 1 million people registered with all 123 primary care practices across three Clinical Commissioning Groups (CCGs) in east London (City and Hackney, Newham and Tower Hamlets). The local authorities covered by these NHS services are among the 10% most socially deprived areas in England, and the ethnically diverse population includes large South Asian and Black British, African and Caribbean ethnic groups.^11^

The review of blood pressure treatment included patients with diagnosed hypertension, and the review of cholesterol-lowering treatment included patients with diagnosed ischaemic CVD (myocardial infarction, angina and other IHD, peripheral artery disease, any stroke or transient ischaemic attack). The extracted data included primary care practice code, individual’s age, sex, ethnicity, Index of Multiple Deprivation (IMD)^11^ quintile, smoking status, measures of total and high-density lipoprotein cholesterol (HDL-C), triglycerides, creatinine, systolic and diastolic blood pressure, prescribed cholesterol-lowering and antihypertensive medications, previous CVD, hypertension, diabetes and chronic kidney disease (CKD). Individual LDL-C levels were calculated using the Friedewald formula.^12^

For a small number of patients, data were missing for IMD, sex, ethnicity, smoking status, cholesterol, creatinine and blood pressure (6.5% for HDL-C, <5% for others). Missing IMD quintiles (0.05%) were assigned to the most populous IMD quintile by primary care practice. Missing ethnicities (2.5%) were assigned to the most common ethnic group for the area. Missing sex (one patient) was assigned to ‘men’, reflecting greater proportion of men in the study population. Other missing values were imputed using multiple imputation with chained equations, including all patient characteristics as covariates.^13^

### The Heart Protection Study CVD policy model

The Heart Protection Study-CVD (HPS-CVD) policy model,^14^ a Markov model employing parametric survival models for CVD endpoints and a linear regression model for annual hospital care costs, developed using the individual participant data of the HPS, was used to project the outcomes in the present study without and with treatment optimisation. The non-vascular mortality rates in the model, originally based on lifetable data for England, was replaced with 2019 mortality data for the three east London CCGs (online supplemental table S1). The original cardiovascular risk equations were adjusted for ethnicity (by sex) in patients without previous CVD (online supplemental table S2)^15^ and calibrated to year 2018 using decreasing CVD trends in England between 2001 and 2018 (online supplemental table S3).^16 17^ Based on patient characteristics at entry, the model projects annual risks of vascular and non-vascular death, non-fatal major vascular event (MVE: myocardial infarction, stroke, arterial revascularisation) and other vascular event (admission for angina, heart failure or other cardiac or vascular problem).

10.1136/bmjopen-2021-052884.supp1 Supplementary data

### Antihypertensive treatment

Optimising antihypertensive treatment was considered for all patients diagnosed with hypertension whose latest blood pressure measurement indicated suboptimal control (ie, systolic/diastolic blood pressure >140/90 mm Hg for those aged <80 years, or >150/90 mm Hg for those aged ≥80 years, according to NICE guidance^6^), irrespective of measurement setting (>99% of blood pressure measures were made in clinic). Blood pressure treatment, including up to three antihypertensive agents, was categorised in line with NICE guidance.^6^

The antihypertensive medications were grouped into angiotensin converting enzyme inhibitors (ACEi)/angiotensin receptor blockers (ARB), calcium channel blockers (CCB), thiazide diuretics (TD) and others (including beta blockers, spironolactone/potassium-sparing diuretics, alpha blockers and loop diuretic). In the optimisation strategy, first-line treatment recommended by NICE was applied for patients who were not using any antihypertensive treatment. TD was added for patients already on the recommended first-line treatment; ACEi/ARB or CCB, respectively, was added (as per indicated first-line treatment) to those on antihypertensive medication different from the recommended first-line recommendation; ACEi/ARB, CCB or TD, respectively, was added for patients already on the other two categories of antihypertensives. No further optimisation was considered for patients who already used a combination of ACEi/ARB, CCB and TD treatment independently of their achieved blood pressure; these patients are regarded as having resistant hypertension^6^ and beyond the scope of this optimisation programme.

Ramipril 5 mg/day was used for ACEi/ARB treatment, amlodipine 5 mg/day for CCB treatment and indapamide 2.5 mg/day for TD treatment with additive effects with use of two or more categories.^18^ These regimens were expected to achieve systolic blood pressure reductions of 7.47 (95% CI 2.19 to 12.76),^19^ 8.9 (7.66 to 10.14)^20^ and 11.94 (7.99 to 15.88)^21^ mm Hg, respectively. A tenth of the difference between preoptimised systolic blood pressure values (mm Hg) and a reference value (154 mm Hg) was deducted from (preoptimised systolic blood pressure <154) or added to (preoptimised systolic blood pressure >154) the expected systolic blood pressure reduction in line with the finding that greater reduction is elicited when treating a higher initial blood pressure (on average, a further 1 mm Hg systolic blood pressure reduction per 10 mm Hg preoptimised systolic blood pressure above 154 mm Hg).^18^ The effects of antihypertensive treatments were estimated using the expected systolic blood pressure reductions and the HRs for reductions in cardiovascular events per mm Hg systolic blood pressure reduction with antihypertensive treatment, reported by the Blood Pressure Lowering Treatment Trialists’ Collaboration (online supplemental table S4).^3 4^

### Cholesterol-lowering treatment with statins

Statin treatment optimisation was considered for patients with history of CVD not receiving statin treatment or receiving suboptimal low or medium-intensity treatment. For such patients, the current NICE guidance recommends starting statin treatment with atorvastatin 80 mg, unless the patient has CKD, there is high risk of adverse effects or alternative preference; the recommended statin treatment is irrespective of starting LDL-C concentration.^7^ To assess the achieved reduction in LDL-C under ongoing treatments, the cholesterol-lowering regimens used by patients were grouped into three intensity levels with expected LDL-C reduction^7^ of ≥45% for high-intensity, 35%–45% for medium-intensity and <35% for low-intensity regimens (see online supplemental table S5 for more details).

The optimisation strategy followed the NICE guidance and used atorvastatin 80 mg in patients aged under 75 years and without CKD, and atorvastatin 40 mg in patients aged 75 years and older or with CKD (online supplemental table S6).^22^ The effects of statin treatments were projected using effects of statin therapy per 1 mmol/L LDL-C reduction, reported by Cholesterol Treatment Trialists’ (CTT) Collaboration meta-analysis of randomised studies (online supplemental table S4).^5^ The effect of statin regimens used prior to optimisation was simulated based on achieved reductions in LDL-C at 1 year in randomised controlled trials included in CTT in categories by low/medium or high-intensity regimens.^5^ Expected further reduction in LDL-C achieved with the optimised statin therapy was determined by the preoptimised LDL-C level and the difference between proportional reductions in LDL-C achieved by the optimised and preoptimised statin regimens (online supplemental table S7).

### Healthcare costs and health-related quality of life

Annual hospital care costs in the HPS model were inflated to 2019 (online supplemental table S8).^23^ The extra medication costs were estimated as the difference between the cost of the optimised treatment and the costs of the respective preoptimisation treatments (online supplemental table S9).^24^

We estimated health-related quality of life (QoL) related to patient characteristics, including experience of cardiovascular events, using a linear regression model of EuroQol-5 dimension utility related to individual characteristics, using data from the Health Surveys for England (online supplemental table S10). The QoL utility values range from −0.594 for the worst health state to 1 for full health, where 0 is a health state equivalent to death and higher values indicate better QoL.^25^ The patients’ predicted QoL during each year in the model was combined with their predicted survival to estimate quality-adjusted life year (QALY).

### Effects of optimisation strategies

The model projected non-fatal MVEs, other non-fatal vascular events, deaths from vascular causes, survival (ie, life years), QALYs, annual hospital care costs and additional medication costs. The added life years and QALYs gained were calculated for three optimisation scenarios: optimising 10% and 20% additional patients from respective overall target populations and optimising all patients not on optimal treatment. The results are presented by age category (<50, 50–59, 60–69, 70–79 and ≥80 years), with projections over 5 years, 10 years and over patients’ lifespans. The parameter uncertainty was assessed using the non-parametric bootstrap approach and 1000 non-parametric bootstrap resamples in HPS model.^14^ The uncertainty in risk ratios of statin and antihypertensive treatments used sampling from their respective lognormal distributions.

While antihypertensive and statin treatments were optimised separately in the two study populations, in a scenario analysis, the effect of optimising both antihypertensive and statin treatments in patients with hypertension and CVD and not on optimal antihypertensive nor optimal statin treatment was evaluated.

**Figure 1:**
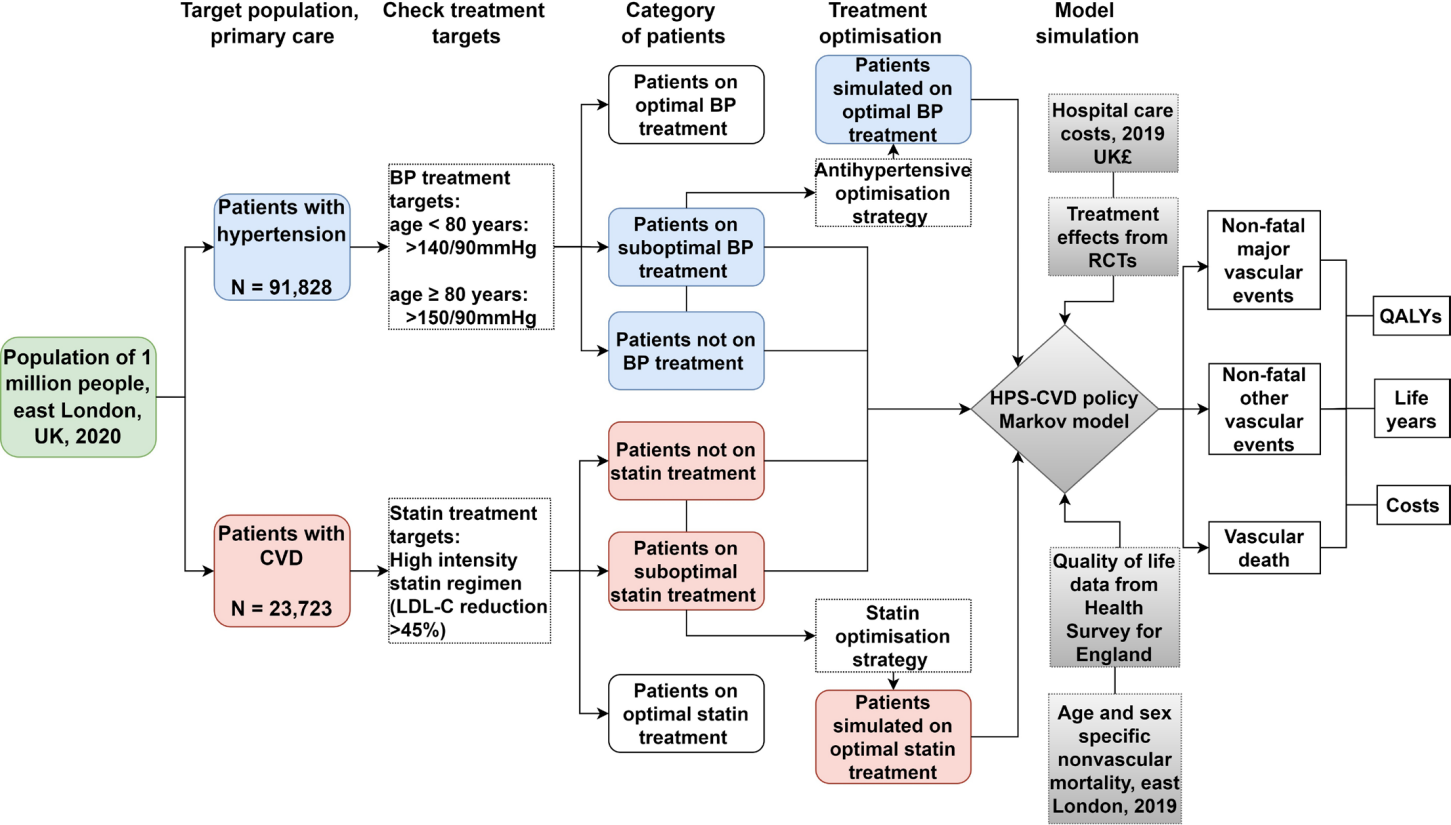
A flowchart of the procedure of the data analysis and model projection. CVD, cardiovascular disease; BP, blood pressure; LDL-C, low-density lipoprotein cholesterol; HPS, Heart Protection Study; QALY, quality-adjusted life year; RCT, randomised controlled trial.

The analytical framework is presented in figure 1. All analyses were performed in R 4.0.2.

## Results

### Baseline characteristics of the study population

There were 91 828 patients diagnosed with hypertension and 23 723 patients with prior CVD in the three east London CCGs. The study population had high levels of deprivation, with 93% of individuals in the bottom two quintiles of the socioeconomic deprivation in England and was highly ethnically diverse (35% white, 31% South Asian and 26% black). Of the patients with diagnosed hypertension, 21 954 (24%) were not on optimal antihypertensive treatment, and in 2867 (3%) patients, the treatment status was unknown due to missing blood pressure measures (table 1). Of patients not on optimal treatment, 18 282 (83%) were on antihypertensive treatment, but their blood pressure was not at target and they were not on triple antihypertensive treatment with combination of ACEi/ARB, CCB and TD, and 3672 (17%) were not treated with any antihypertensive medication (table 1 and online supplemental tables S11,12). Of the patients with prior CVD, 9062 (38%) were not on optimal cholesterol-lowering treatment (table 1), of whom, 5729 (63%) were on suboptimal low or medium-intensity cholesterol-lowering treatment, and 3333 (37%) were untreated (table 1, online supplemental tables S5, S12).

**Table 1:** Characteristics of patients with diagnosed hypertension or prior cardiovascular disease in the three east London Clinical Commissioning Groups

	Patients with diagnosis of hypertension			Patients with prior cardiovascular disease		
	Total‡	Antihypertensive treatment		Total		Cholesterol-lowering treatment
		Optimal§	Suboptimal/untreated¶		Optimal	Suboptimal/untreated**
N (%)	**91 828**	**67 007** (**73%**)	**21 954** (**24%**)	23 723	**14 661** (**62%**)	**9062** (**38%**)
CCG*†						
City and Hackney	27 641 (30%)	20 722 (31%)	6251 (28%)	7204 (30%)	4614 (31%)	2590 (29%)
Newham	40 287 (44%)	27 691 (41%)	10 820 (49%)	9417 (40%)	5137 (35%)	4280 (47%)
Tower Hamlets	23 900 (26%)	18 594 (28%)	4883 (22%)	7102 (30%)	4910 (33%)	2192 (24%)
Age*†	62.3 (13.7)	63.6 (13.7)	59.1 (13.1)	67.5 (13)	66.4 (12.2)	69.3 (14.0)
Sex*†						
Female	47 184 (51%)	35 211 (53%)	10 827 (49%)	8918 (38%)	4993 (34%)	3925 (43%)
Male	44 643 (49%)	31 795 (47%)	11 127 (51%)	14 805 (62%)	9668 (66%)	5137 (57%)
Missing	1	1	0	0	0	0
Ethnicity*†						
White	31 340 (34%)	23 109 (34%)	7179 (33%)	10 265 (43%)	6150 (42%)	4115 (45%)
Black	24 976 (27%)	17 326 (26%)	6810 (31%)	3573 (15%)	2024 (14%)	1549 (17%)
South Asian	28 277 (31%)	21 342 (32%)	6315 (29%)	8246 (35%)	5529 (38%)	2717 (30%)
Other	4982 (5.4%)	3789 (5.7%)	1019 (4.6%)	1091 (4.6%)	638 (4.4%)	453 (5.0%)
Missing	2253 (2.5%)	1441 (2.2%)	631 (2.9%)	548 (2.3%)	320 (2.2%)	228 (2.5%)
IMD quintile*†						
Q1 (least deprived)	604 (0.66%)	469 (0.7%)	118 (0.54%)	170 (0.72%)	103 (0.70%)	67 (0.74%)
Q2	1241 (1.4%)	918 (1.4%)	269 (1.2%)	318 (1.3%)	181 (1.2%)	137 (1.5%)
Q3	4237 (4.6%)	3118 (4.7%)	979 (4.5%)	1073 (4.5%)	637 (4.3%)	436 (4.8%)
Q4	36 810 (40%)	26 498 (40%)	9013 (41%)	9335 (39%)	5536 (38%)	3799 (42%)
Q5 (most deprived)	48 894 (53%)	35 974 (54%)	11 566 (53%)	12 818 (54%)	8202 (56%)	4616 (51%)
Missing	42 (0.05%)	30 (0.04%)	9 (0.04%)	9 (0.04%)	2 (0.01%)	7 (0.08%)
Smoking status*†						
Non-smoker	58 994 (64%)	43 031 (64%)	14 037 (64%)	11 434 (48%)	6645 (45%)	4789 (53%)
Ex-smoker	19 859 (22%)	15 062 (22%)	4355 (20%)	7597 (32%)	4969 (34%)	2628 (29%)
Current smoker	12 536 (14%)	8679 (13%)	3412 (16%)	4622 (19%)	3015 (21%)	1607 (18%)
Missing	439 (0.48%)	235 (0.35%)	150 (0.68%)	70 (0.30%)	32 (0.22%)	38 (0.42%)
Diseases status						
Myocardial infarction*†	3725 (4.1%)	3031 (4.5%)	668 (3.0%)	6284 (26%)	4739 (32%)	1545 (17%)
Angina*†	3390 (3.7%)	2771 (4.1%)	604 (2.8%)	4761 (20%)	3017 (21%)	1744 (19%)
Other IHD*†	9408 (10%)	7654 (11%)	1699 (7.7%)	14 303 (60%)	9381 (64%)	4922 (54%)
PAD*†	1799 (2.0%)	1444 (2.2%)	347 (1.6%)	2627 (11%)	1526 (10%)	1101 (12%)
Stroke*†	5439 (5.9%)	4370 (6.5%)	1019 (4.6%)	7744 (33%)	4303 (29%)	3441 (38%)
Atrial fibrillation*†	4171 (4.5%)	3450 (5.1%)	684 (3.1%)	2462 (10%)	1418 (10%)	1044 (12%)
Heart failure*†	3852 (4.2%)	3152 (4.7%)	671 (3.1%)	3026 (13%)	1985 (14%)	1041 (11%)
Diabetes*†	32 716 (36%)	26 524 (40%)	5949 (27%)	10 344 (44%)	7077 (48%)	3267 (36%)
CKD*†	16 423 (18%)	13 191 (20%)	3058 (14%)	6279 (26%)	3661 (25%)	2618 (29%)
Hypertension				15 442 (65%)	9508 (65%)	5934 (65%)
Clinical measures						
Total cholesterol (mmol/L)*†	4.5 (1.2)	4.3 (1.1)	4.7 (1.2)	4.0 (1.2)	3.8 (1.2)	4.3 (1.1)
Triglycerides (mmol/L)*†	1.5 (1.1)	1.5 (1.1)	1.5 (1.1)	1.6 (1.09)	1.6 (1.16)	1.5 (0.94)
HDL-C (mmol/L)*†	1.4 (0.38)	1.4 (0.38)	1.4 (0.39)	1.3 (0.37)	1.3 (0.35)	1.4 (0.38)
Creatinine (μmol/L)	90 (59)	89 (56)	90 (66)	98 (66)	97 (63)	99 (71)
Systolic blood pressure (mm Hg)*†	133 (14)	128 (10)	147 (13)	128 (15)	128 (15)	130 (15)
Diastolic blood pressure (mm Hg)*†	78 (10)	76 (8)	87 (11)	74 (10)	74 (10)	75 (10)

Column % presented for totals and, separately, for patients on optimal and not optimal treatment.

For categorical variables, Χ^2^ test was conducted for the pairs of categories of interest: black versus white (antihypertensive p<0.001; statin p<0.01); black versus south Asian (antihypertensive p<0.001; statin p<0.001); IMD Q5 versus Q1-4 (antihypertensive p<0.01; statin p<0.001).

*p<0.05 for difference between patients on optimal and suboptimal/untreated antihypertensive treatment.

†p<0.05 for difference between patients on optimal versus suboptimal/untreated statin treatment.

‡Includes 2867 (3%) patients with diagnosis of hypertension but unknown antihypertensive management status due to missing blood pressure measure.

§Includes 3584 patients with blood pressure not on target who are already on three-agent antihypertensive treatment (resistant hypertension).

¶Includes 18 282 suboptimally treated and 3672 untreated patients.

**Includes 5729 suboptimally treated and 3333 untreated patients.

CCG, Clinical Commissioning Groups; IMD, index of multiple deprivation; IHD, ischaemic heart disease; PAD, peripheral artery disease; CKD, chronic kidney disease; HDL-C, high density lipoprotein cholesterol.

Among patients with diagnosed hypertension, men were less likely to be on optimal antihypertensive treatment compared with women (74% vs 76%; p<0.001), as were patients of black (72%) compared with white (76%; p<0.001) or South Asian (77%; p<0.001) ethnicities (table 1). Among patients with previous CVD, women were less likely to be on optimal cholesterol-lowering treatment compared with men (56% vs 65%; p<0.001), as were patients of black ethnicity (57%) compared with white (60%; p<0.01) or South Asian (67%; p<0.001) ethnicities. Patients with diagnosed hypertension not on optimal antihypertensive treatment were younger (mean age 59 vs 64 years; p<0.001), while patients with previous CVD not on optimal cholesterol-lowering treatment were older (69 vs 66 years; p<0.001). Compared with all other socioeconomic quintiles together, patients in the most deprived quintile were more likely to be on optimal antihypertensive (76% vs 75%; p<0.01) and cholesterol-lowering (64% vs 59%; p<0.001) treatment.

### Projected health benefits with optimised treatments

Optimising treatments was projected to substantially reduce CVD risks (table 2). Optimising antihypertensive treatment in 1000 patients with hypertension was evaluated to lead to 25 (95% CI 16 to 32) fewer non-fatal MVEs and 7 (3 to 10) fewer vascular deaths in 5 years, and 151 (72 to 223) fewer non-fatal MVEs and 65 (27 to 98) fewer vascular deaths over patients’ lifetimes. Similarly, the optimisation of statin treatment in 1000 patients with CVD was projected to lead to 28 (22 to 33) fewer non-fatal MVEs and 6 (4 to 7) fewer vascular deaths in 5 years, and 139 (100 to 173) fewer non-fatal MVEs and 31 (22 to 38) fewer vascular deaths over patient lifetimes.

**Table 2:** Predicted reductions in cardiovascular events with optimised antihypertensive or statin treatment

Time horizon	Cardiovascular events avoided with optimised antihypertensive or statin treatment		
	Non-fatal MVEs avoided (per 1000 treated) (95% CI)	Non-fatal OVEs avoided (per 1000 treated) (95% CI)	Vascular deaths avoided (per 1000 treated) (95% CI)
Patients with hypertension not on optimal antihypertensive treatment (N=22 191)*			
5 years	25 (16 to 32)	32 (24 to 40)	7 (3 to 10)
10 years	50 (31 to 67)	67 (49 to 83)	15 (6 to 22)
Lifetime	151 (72 to 223)	209 (140 to 274)	65 (27 to 98)
Patients with prior CVD not on optimal statin treatment (N=9062)			
5 years	28 (22 to 33)	15 (4 to 26)	6 (4 to 7)
10 years	54 (42 to 65)	29 (8 to 50)	12 (9 to 15)
Lifetime	139 (100 to 173)	64 (2 to 124)	31 (22 to 38)

Only one vascular event is simulated during each year in the model with priority given to more severe events.

MVE, major vascular events, defined as non-fatal myocardial infarction or death from coronary disease, any stroke, or revascularisation procedure. OVE, other vascular event, defined as admission for angina, heart failure, or other cardiac or vascular problem.

*237 patients with hypertension and unknown blood pressure were categorised into this group following missing blood pressure multiple imputation.

CVD, cardiovascular disease.

Optimising antihypertensive and, separately, statin treatments was predicted to importantly improve life expectancy and QALYs over patient lifetimes (table 3). The predicted QALY gains from optimised antihypertensive treatment in patients with hypertension ranged from 1.11 (95% CI 0.64 to 1.49) QALYs for those aged under 50 years to 0.31 (0.17 to 0.43) QALYs for those aged 80 years or over, with 0.24 (0.12 to 0.33) MVEs and 0.05 (0.00 to 0.09) MVEs avoided, respectively (online supplemental table S13). The estimated QALY gains for patients with CVD from optimised statin treatment ranged from 0.72 (0.55 to 0.90) QALY for those aged under 50 years to 0.12 (0.09 to 0.14) QALYs for those aged 80 years or over, with 0.34 (0.25 to 0.42) and 0.05 (0.04 to 0.07) MVEs avoided, respectively (online supplemental table S13). Optimising antihypertensive and statin treatments was also predicted to reduce hospital care costs (table 3). Both health benefits and cost savings were larger among younger patients.

**Table 3:** Predicted lifetime gains in survival and QALYs, hospital care cost savings, and additional medication cost of fully optimised antihypertensive and statin treatment

Age (years)	Patients with hypertension not on optimal antihypertensive treatment	Patients with prior CVD not on optimal statin treatment
	N=22 191	N=9062
Life years gained per optimised patient (95% CI)		
<50	1.36 (0.57 to 2.04)	1.03 (0.74 to 1.32)
50–59	1.08 (0.45 to 1.61)	0.72 (0.52 to 0.91)
60–69	0.91 (0.38 to 1.36)	0.47 (0.34 to 0.59)
70–79	0.66 (0.27 to 0.98)	0.30 (0.22 to 0.37)
≥80	0.54 (0.22 to 0.80)	0.22 (0.16 to 0.27)
QALYs gained per optimised patient (95% CI)		
<50	1.11 (0.64 to 1.49)	0.72 (0.55 to 0.9)
50–59	0.83 (0.47 to 1.12)	0.49 (0.37 to 0.59)
60–69	0.64 (0.36 to 0.87)	0.30 (0.24 to 0.37)
70–79	0.43 (0.24 to 0.59)	0.18 (0.14 to 0.22)
≥80	0.31 (0.17 to 0.43)	0.12 (0.09 to 0.14)
Hospital care cost savings (£) per optimised patient (95% CI)		
<50	3100 (1040 to 5092)	3508 (2432 to 4472)
50–59	2058 (417 to 3653)	2272 (1494 to 2978)
60–69	1448 (36 to 2879)	1373 (862 to 1841)
70–79	831 (-224 to 1910)	751 (414 to 1082)
≥80	353 (-552 to 1242)	390 (146 to 629)
Extra medication costs (£) per optimised patient (95% CI)		
<50	712 (692 to 728)	728 (712 to 744)
50–59	521 (507 to 533)	406 (395 to 416)
60–69	389 (377 to 399)	248 (241 to 254)
70–79	267 (258 to 274)	114 (110 to 117)
≥80	159 (152 to 165)	54 (52 to 56)

1203 patients with hypertension and previous CVD included in both patient categories.

CVD, cardiovascular disease; QALY, quality-adjusted life year.

### Projected programme impacts

Optimisation of antihypertensive treatment in all patients with hypertension in the three east London CCGs was predicted to lead to 22 228 (95% CI 9234 to 33 296) more life years, 16 698 (9485 to 22 482) more QALYs, and to save £41 069 942 (6 894 396 to 74 751 579) in hospital care costs (online supplemental table S14, figure S1). Similarly, optimisation of statin treatment in all patients with prior CVD in the three east London CCGs was predicted to lead to 4034 (2948 to 5059) more life years, 2616 (2022 to 3184) more QALYs, and to save £11 603 287 (7 243 739 to 15 597 438) in hospital care costs.

**Figure 2:**
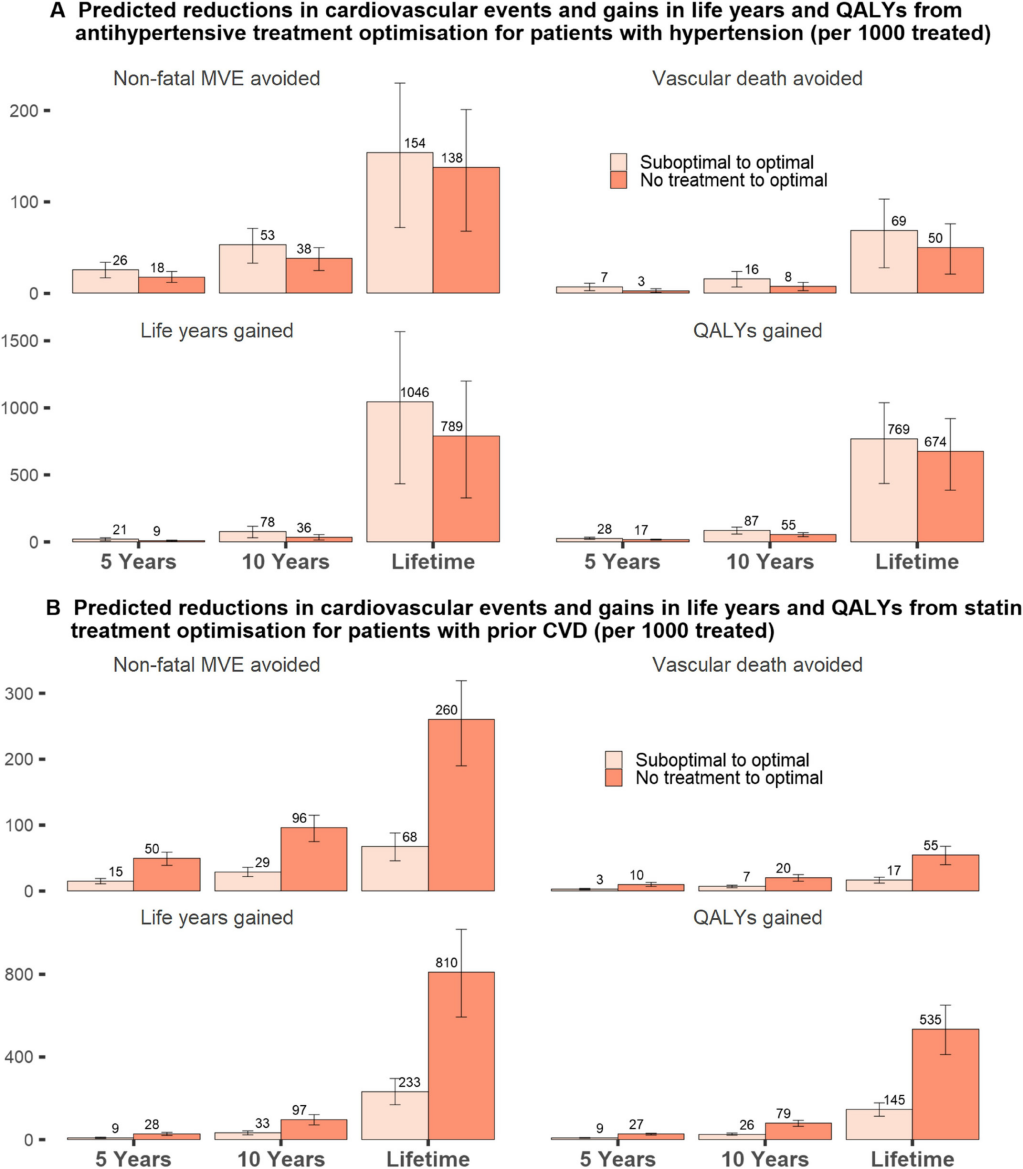
Model projected benefits from antihypertensive and statin treatment optimisation for patients previously on suboptimal treatment or not treated. CVD, cardiovascular disease; MVE, major vascular event; QALY, quality-adjusted life year.

The benefits from optimising suboptimal antihypertensive treatments were larger than those from optimising those not on antihypertensive treatment, while the opposite was true with optimising statin treatment, where larger benefits were projected among patients not on cholesterol-lowering treatment (figure 2, online supplemental table S15). Optimising both antihypertensive and statin treatments in patients needing both optimised, resulted in complementary gains in life years, QALYs and hospital care cost saving (online supplemental table S16).

### Scenario analyses

Given that 73% of the patients with diagnosed hypertension were on optimal antihypertensive treatment before treatment optimisation, a 10% increase in 83% required 9183 extra patients to be optimised. Over lifetime, this scenario was projected to achieve 1393 fewer non-fatal MVEs and 601 fewer vascular deaths, respectively, and to add 9220 life years and 6925 QALYs, respectively (online supplemental figure S1). A 10% increase in the proportion of the patients with CVD on optimal statin treatment (ie, from 62% to 72%) required treatment optimisation in 2372 additional patients. Over lifetime, this scenario was estimated to lead to 326 fewer non-fatal MVEs and 72 fewer vascular deaths and increase life years by 1044 and QALYs by 683, respectively (online supplemental figure S1). The benefits were doubled by a 20% increase in the optimised patients (online supplemental figure S1).

## Discussion

This analysis of primary care records of a large inner city population in London, UK indicates that 27% of patients with hypertension and 38% of patients with CVD do not receive optimal antihypertensive and cholesterol-lowering treatments, respectively. The subsequent projections, using a CVD model, suggest that optimising the use of these treatments will achieve substantial further reductions in cardiovascular events and gains in survival and QoL while also reducing hospital care costs with larger benefits achieved among younger patients. Moreover, given the concentration of hypertension and CVD among people in the highest categories of socioeconomic deprivation, and the disproportionately high percentage of suboptimal treatment among patients of black ethnicity, the optimisation of cardiovascular prevention treatments will also reduce health inequalities.

Despite study population situated among local authorities in the highest socioeconomic deprivation decile in England,^11^ the proportions of optimally managed patients in our study are high, both compared with other local authorities in England,^26^ and to European and worldwide data^9 27^ and were not adversely associated with level of deprivation. This has been achieved over 20 years of locally sustained, digitally supported and incentivised quality improvement programmes.^28^ However, our data suggest that there is still scope for improvement in management of blood pressure and cholesterol, and the improved understanding of where the gaps are will inform strategic choices. In the present study, patients not on optimal antihypertensive treatment, most without previous CVD, were more likely to be younger and men, while those not optimally treated with statin, in a population with previous CVD, were older and women. Patients of black ethnicity were also more likely to require treatment optimisation. Similar demographic and ethnic associations with suboptimal use of cardiovascular prevention medications have been previously reported^9 29^ and point to a need for action.

The benefits from optimising treatments to reduce CVD accrue over patient lifetimes and vary across categories of patients. Differences in characteristics of patients requiring optimisation lead to different sizes of benefits from optimisation, which, in our study, are larger in younger and predominantly without previous CVD population requiring optimisation of blood pressure, compared with the population with history of CVD requiring statin treatment optimisation. Despite variation in size of benefits, however, successful treatment optimisation is shown to be highly beneficial across all patient categories studied.

Our study indicates that there are differences between suboptimally treated and untreated individuals in terms of mean age, comorbidities and other risk factors; these differences also vary between the two treatments studied. Among individuals with increased blood pressure, optimising suboptimal treatment resulted in larger benefits compared with optimising untreated patients because of higher disease risks in this category, and also because effects of antihypertensive treatments are additive.^18^ The opposite was the case among people with CVD, where benefits from optimising statin treatment were much larger among untreated compared with suboptimally treated, mainly because increasing treatment intensity, for example, doubling of statin dose, produces only 6% reduction in LDL cholesterol.^30^ This study falls short, however, of evaluating cost-effectiveness of programmes to optimise cardiovascular prevention in primary care. Such evaluations require estimates of programme’s impact on uptake and adherence to optimal treatment as well as the cost of programme implementation and management. Interventions to improve adherence to statin and blood pressure treatments have been an area of active research, though with limited success so far.^31 32^ Complex interventions, combining elements targeting knowledge and behaviour of patients and clinicians and facilitation from the health system, have shown some promise, and the projections reported in the present study can facilitate the evaluation of cost-effectiveness of such programmes.

Our study has some further limitations. First, while we adapted and calibrated the HPS-CVD model for use in the present study, these adjustments were based on published estimates and population statistics for England rather than data specific to the study population. Second, we present results under full adherence to optimal treatments to motivate action; suboptimal adherence, however, may erode the benefits projected here. Third, we did not account for adverse effects of antihypertensive and statin treatments alone or in combination with concomitant medications, which may result in drug withdrawal and reductions in QALYs. Fourth, while we report the cost for additional blood pressure and statin medications, we did not account for any additional consultations and testing in primary care to initiate and support people on treatment. There is, however, ongoing effort in UK primary care to optimise monitoring and management across individual’s needs and, therefore, extra consultations may not be needed.^33^ Finally, this study only included patients with hypertension and previous CVD and focused on blood-pressure lowering and statin treatments; future investigations could be extended to optimising statin treatment for primary CVD prevention or indeed consider the use of novel interventions, such as PCSK9 inhibitors, to further reduce population CVD risks.^34^

In conclusion, our paper presents a comprehensive population study in 1 million people in east London, an urban area with high ethnic diversity and high deprivation. We report gaps in antihypertensive treatment among patients with hypertension and in statin treatment among patients with previous CVD. Optimising these treatments will reduce vascular events, increase life expectancy and QALYs and reduce hospital care costs while also reducing socioeconomic inequalities in health. Developments of programmes to bring forward improvements in initiation and adherence to cardiovascular preventive interventions need to be prioritised.

## Data Availability

All data relevant to the study are included in the article or uploaded as supplementary information. All general practitioners in the participating east London practices consented to the use of their anonymised patient data for research and development for patient benefit.
