# Supplementary material for: Gaps in antihypertensive and statin treatments and benefits of optimisation: a modelling study in a 1 million ethnically diverse urban population in UK
Source: BMJ Open. 2021 Dec 30;11(12):e052884. doi: 10.1136/bmjopen-2021-052884 (PMC8719215; doi:10.1136/bmjopen-2021-052884)
Supplement: Supplementary data [file bmjopen-2021-052884supp001.pdf]

Gaps in antihypertensive and statin treatments and benefits of optimisation: a modelling study in a 1 million ethnically diverse urban population in UK

Supplementary Material

Supplementary Table S1: 2019 age- and sex- specific nonvascular mortality rates in the London boroughs of City, Hackney, Newham and Tower Hamlets

Age (years)	Population (N)	All deaths	Cardiovascular deaths	Nonvascular deaths	Nonvascular mortality rate	Probability of nonvascular death
			Men			
15-24	62,821	29	0	29	0.000461629	0.000461523
25-34	121,088	47	0	47	0.000388147	0.000388072
35-44	94,063	73	15	58	0.000616608	0.000616418
45-54	56,107	176	58	118	0.002103124	0.002100914
55-64	36,620	258	92	166	0.004533042	0.004522783
65-74	19,161	370	98	272	0.014195501	0.01409522
75-84	9,740	464	128	336	0.03449692	0.033908685
85+	3,631	428	96	332	0.091434866	0.087379244
			Women			
15-24	61,660	5	0	5	0.00008109	0.000081087
25-34	109,119	21	0	21	0.00019245	0.000192432
35-44	76,083	49	5	44	0.000578316	0.000578149
45-54	50,573	95	12	83	0.001641192	0.001639846
55-64	36,296	171	36	135	0.003719418	0.00371251
65-74	22,102	262	43	219	0.009908606	0.009859677
75-84	11,740	400	100	300	0.025553663	0.025229931
85+	5,047	538	142	396	0.078462453	0.075463227

Data sourced from Office for National Statistics[1 2]. Annual probability of nonvascular death is 1-exp(-nonvascular mortality rate).

Supplementary Table S2: Cardiovascular risk calibrating parameters for patients without previous cardiovascular disease

Ethnicity	Men<br>Hazard Ratio (95% CI)	Women<br>Hazard Ratio (95%CI)
White	1	1
Black	0.68 (0.64, 0.73)	0.71 (0.66, 0.77)
South Asian	1.48 (1.42, 1.55)	1.43 (1.35, 1.51)
Other	0.82 (0.76, 0.87)	0.87 (0.80, 0.95)

Source: Hippisley-Cox, et al.[3]

Supplementary Table S3: CVD mortality and incidence rates (per 100,000) in England

	CVD mortality	CVD morbidity[1]
2001	153.4	NA
2002	145.6	NA
2003	138.2	NA
2004	127.5	NA
2005	119.1	1026.0
2006	111.6	986.6
2007	104.9	940.5
2008	99.5	918.2
2009	92.7	899.3
2010	90.4	878.8
2011	82.6	914.8
2012	79.3	925.6
2013	78.1	894.6
2014	75.6	892.2
2015	75.7	889.8
2016	74.4	877.7
2017	72.7	851.4
2018	73.4	NA

NA, not available.
Sources: British Heart Foundation 2020.[4]
[1]CVD (cardiovascular disease) incidence (fatal and nonfatal) includes atrial fibrillation, heart failure, stroke, transient ischemia attack, and peripheral vascular disease. Only data for 2005-2017 are available.
The calibration factors are calculated as follows:

Calibration factor (CVD death)= 2018 CVD mortality rate/2001 CVD mortality rate = 47.9%

Therefore, the reduction in CVD death from 2001 to 2018 was 52.1%. The reduction in CVD death from 2005 to 2017 was 1-2017 CVD mortality rate/2005 CVD mortality rate, or 39% which is 74.8% of the 52.1% reduction from 2001 to 2018.

We assume that the reduction in CVD morbidity was following similar trends. The reduction in CVD morbidity from 2005 to 2017 was 1- 2017 CVD morbidity rate/2005 CVD morbidity rate, or 17%. If this is 74.8% of reduction in CVD morbidity from 2001 to 2018 then the overall reduction is 17%/74.8%

Calibration factor (CVD morbidity) = 1 - 17%/ 74.8% = 77.2%

Wu R, *et al. BMJ Open* 2021; 11:e052884. doi: 10.1136/bmjopen-2021-052884

Supplementary Table S4: Effects of antihypertensive and statin treatments

	Risk Ratio (95% CI)
**Antihypertensive treatment[1]**	
Vascular death (per 4.5 mm Hg reduction)	0.85 (0.76, 0.95)
All vascular event or vascular death (per 5 mm Hg reduction)	0.83 (0.79, 0.87)
**Statin treatment (per 1 mmol/L LDL-C reduction)[2]**	
Vascular death	0.86 (0.82, 0.90)
Major vascular event or vascular death	0.77 (0.74, 0.82)
Any vascular event or vascular death	0.84 (0.81, 0.87)

Sources: [1]Blood Pressure Lowering Treatment Trialists' Collaboration[5 6]; [2]Cholesterol Treatment Trialists' Collaboration[7]

Supplementary Table S5: Cholesterol-lowering treatment regimens among patients with prior cardiovascular disease (N =23723)

Cholesterol-lowering treatment	Number patients	Expected LDL-C reduction[1]	Cholesterol-lowering Intensity[2]
Atorvastatin 10mg	579	37%	Medium
Atorvastatin 10mg+Ezetimibe 10mg	5	53%	High
Atorvastatin 20mg	2286	43%	Medium
Atorvastatin 20mg+Ezetimibe 10mg	30	54%	High
Atorvastatin 40mg	6162	49%	High
Atorvastatin 40mg+Ezetimibe 10mg	83	56%	High
Atorvastatin 60mg	4	52%	High
Atorvastatin 80mg	7927	55%	High
Atorvastatin 80mg+Ezetimibe 10mg	201	61%	High
Fluvastatin 20mg	3	21%	Low
Fluvastatin 20mg+Ezetimibe 10mg	1	32%	Low
Fluvastatin 40mg	5	27%	Low
Fluvastatin 40mg+Ezetimibe 10mg	1	37.5%	Medium
Pravastatin 10mg	50	20%	Low
Pravastatin 20mg	83	24%	Low
Pravastatin 40mg	119	29%	Low
Pravastatin 40mg+Ezetimibe 10mg	8	41%	Medium
Rosuvastatin 5mg	173	38%	Medium
Rosuvastatin 5mg+Ezetimibe 10mg	13	52.8%	High
Rosuvastatin 10mg	167	43%	Medium
Rosuvastatin 10mg+Ezetimibe 10mg	15	59.7%	High
Rosuvastatin 20mg	124	48%	High
Rosuvastatin 20mg+Ezetimibe 10mg	17	63.5%	High
Rosuvastatin 40mg	39	53%	High
Rosuvastatin 40mg+Ezetimibe 10mg	20	70%	High
Simvastatin 10mg	42	27%	Low
Simvastatin 20mg	627	32%	Low
Simvastatin 20mg+Ezetimibe 10mg	9	50.6%	High
Simvastatin 40mg	1345	37%	Medium
Simvastatin 40mg+Ezetimibe 10mg	10	57.4%	High
Simvastatin 80mg	106	42%	Medium
Simvastatin 80mg+Ezetimibe 10mg	2	58.6%	High
Ezetimibe 10mg	134	18.5%	Low
**Subtotal**	**20390 (5729 statin treatment suboptimal)**		
No cholesterol-lowering treatment	3333		
**Total**	**23723**		
**Total for statin treatment optimisation**	**9062**		

[1]Based on NICE guideline and dose-response studies [8-15].

[2]The regimens were categorised according average proportional reduction in LDL cholesterol achieved, with regimens achieving 45% or higher reduction considered high intensity; 35% - 45% medium intensity; and less than 35% low intensity.

Wu R, *et al. BMJ Open* 2021; 11:e052884. doi: 10.1136/bmjopen-2021-052884

Supplementary Table S6: Strategy for optimising statin treatment of patients with prior cardiovascular disease (N = 23723)

<table>
<tr><th rowspan="4">Pre-optimisation statin therapy</th><th colspan="3">Statin optimisation strategy</th></tr>
<tr><th rowspan="2">Patients with CKD</th><th colspan="2">Patients without CKD</th></tr>
<tr><th>≥75 years old</th><th><75 years old</th></tr>
<tr><th>6279</th><th>3893</th><th>13551</th></tr>
<tr><td>High intensity</td><td colspan="2">5728 (no change)</td><td>8933 (no change)</td></tr>
<tr><td>Low/medium intensity</td><td colspan="2">3041 (to atorvastatin 40mg)</td><td>2688 (to atorvastatin 80mg)</td></tr>
<tr><td>No treatment</td><td colspan="2">1403 (to atorvastatin 40mg)</td><td>1930 (to atorvastatin 80mg)</td></tr>
</table>

CKD, chronic kidney disease.

High intensity therapy defined as the statin/ezetimibe regimens expected to achieve LDL cholesterol (LDL-C) reduction of 45% or higher. Low/medium intensity therapy defined as the statin/ezetimibe regimens expected to achieve LDL-C reduction less than 45%.

Wu R, *et al. BMJ Open* 2021; 11:e052884. doi: 10.1136/bmjopen-2021-052884

Supplementary Table S7: LDL-C reduction with statin optimisation

Statin optimisation strategy	LDL-C pre-optimisation (mmol/L) Mean (SD)	Calculating LDL-C reduction with statin optimisation	LDL-C reduction (mmol/L) with statin optimisation Mean (SD)
No change in statin treatment required	2.22(1.03)	NA	NA
No statin treatment to atorvastatin 40mg	3.07 (1.02)	LDL-C pre-optimisation * 49%	1.50 (0.50)
No statin treatment to atorvastatin 80mg	3.16 (1.01)	LDL-C pre-optimisation * 55%	1.74 (0.56)
Low/medium intensity statin to atorvastatin 40mg	2.21 (0.86)	LDL-C pre-optimisation /(1 – % LDL-C reduction with previous statin therapy) * (49% – % LDL reduction with previous statin treatment)	0.39 (0.26)
Low/medium intensity statin to atorvastatin 80mg	2.43 (0.97)	LDL-C pre-optimisation /(1 – % LDL-C reduction with previous statin therapy) * (55% – % LDL-C reduction with previous statin treatment)	0.65 (0.33)

LDL-C, LDL cholesterol.
High intensity therapy defined as the statin/ezetimibe regimens expected to achieve LDL-C reduction of 45% or higher. Low/medium intensity therapy defined as the statin/ezetimibe regimens expected to achieve LDL-C reduction less than 45%.
For example, for the patient currently treated with atorvastatin 10mg, which is expected to reduce LDL-C by 37%, with an LDL-C of 3.5 mmol/L, a statin treatment change to atorvastatin 80mg will achieve an extra LDL-C reduction of 3.5/(1 – 0.37)*(0.55 – 0.37) = 1 mmol/L

Wu R, *et al. BMJ Open* 2021; 11:e052884. doi: 10.1136/bmjopen-2021-052884

Supplementary Table S8: Linear regression model of annual hospital care costs (2019 UK£)

Patient characteristics	Annual hospital care cost[1] (2019 UK£)
Intercept	445 (384, 505)
Female (Ref: Male)	121 (182, 60)
Peripheral arterial disease, stroke or diabetes (Ref: None)	
One	67 (15, 120)
Two or three	254 (156, 353)
MVE in current year	9597 (9215, 9979)
OVE only in current year	4705 (4484, 4926)
MVE or OVE in 1+ year	297 (234, 359)
Non-vascular death	5922 (5393, 6451)
Vascular death	3593 (3190, 3996)
Age (centred at 64; per year)	18 (15, 21)

MVE, major vascular event; OVE, other vascular event.

[1]Based on Heart Protection Study Collaborative Group with costs inflated to 2019 UK£ using the NHS cost inflation index[16].

Wu R, *et al. BMJ Open* 2021; 11:e052884. doi: 10.1136/bmjopen-2021-052884

Supplementary Table S9: Annual medication costs, 2019

Treatment	Annual costs (2019 UK£)[1]
Atorvastatin 10mg tablets	10.05
Atorvastatin 20mg tablets	11.84
Atorvastatin 40mg tablets	14.38
Atorvastatin 80mg tablets	22.50
Ezetimibe 10mg tablets	26.85
Fluvastatin 20mg capsules	27.92
Fluvastatin 40mg capsules	31.35
Fluvastatin 80mg modified-release tablets	250.29
Pravastatin 10mg tablets	10.71
Pravastatin 20mg tablets	13.20
Pravastatin 40mg tablets	16.28
Rosuvastatin 10mg tablets	19.07
Rosuvastatin 5mg tablets	19.02
Simvastatin 10mg tablets	8.93
Simvastatin 20mg tablets	10.65
Simvastatin 40mg tablets	11.80
Simvastatin 80mg tablets	20.09
Ramipril 5mg capsules[2]	12.13
Amlodipine 5mg tablets[2]	9.86
Indapamide 2.5mg tablets[2]	23.54

[1]NHS Drug Tariff obtained from the Prescription Cost Analysis for England 2019, NHS Business Services Authority [17]; [2]Most frequently prescribed in England hypertensive regimens for ACEi/ARB treatment , CCB treatment and TD treatment, respectively (Source: Prescription Cost Analysis for England 2019)

Supplementary Table S10: Linear regression model of individual's health-related quality of life (QoL)

Patient characteristics	*QoL utility[1]*
Intercept	0.97 (0.96, 0.98)
Baseline characteristics	
Female (Ref: Male)	-0.04 (-0.04 , -0.03)
Ethnicity (Ref: White)	
Black	0.00 (-0.01 , 0.01)
South Asian	-0.04 (-0.05 , -0.03)
Other ethnicity	0.01 (-0.01 , 0.02)
IMD (Ref: Quintile 1, least deprived)	
IMD Q2	-0.02 (-0.03 , -0.01)
IMD Q3	-0.03 (-0.04 , -0.02)
IMD Q4	-0.06 (-0.07 , -0.05)
IMD Quintile 5 (most deprived)	-0.11 (-0.12 , -0.10)
Smoking status (Ref: Non-smoker)	
Ex-smoker	-0.02 (-0.02 , -0.01)
Current smoker	-0.05 (-0.06 , -0.04)
Treated Hypertension	-0.04 (-0.05 , -0.03)
Diabetes	-0.09 (-0.10 , -0.07)
MVE in current year	-0.19 (-0.21 , -0.17)
OVE only in current year	-0.09 (-0.11 , -0.08)
MVE in 1+ year	-0.09 (-0.11 , -0.08)
OVE only in 1+ year	-0.04 (-0.06 , -0.01)
Age category (Ref: 40-44 years)	
Age 30-34	0.04 (0.03, 0.05)
Age 35-39	0.02 (0.01, 0.03)
Age 45-49	-0.01 (-0.02, 0.00)
Age 50-54	-0.04 (-0.05, -0.03)
Age 55-59	-0.05 (-0.07, -0.04)
Age 60-64	-0.06 (-0.07, -0.05)
Age 65-69	-0.05 (-0.06, -0.04)
Age 70-74	-0.06 (-0.08, -0.05)
Age 75-79	-0.08 (-0.10, -0.07)
Age 80-84	-0.14 (-0.16, -0.13)
Age 85-89	-0.18 (-0.20, -0.15)
Age 90+	-0.23 (-0.27, -0.19)

QoL, Quality of life; IMD, index of multiple deprivation; CVD, cardiovascular disease; MVE, major vascular event; OVE, other vascular event.

[1]Linear regression using Health Survey for England 2003, 2004, 2006 and 2011 participant data including EuroQoL EQ-5D-3L questionnaire data.

Supplementary Table S11: Patients with diagnosis of hypertension, antihypertensive regimens prior to optimisation, and optimisation strategy for antihypertensive treatment (N = 91828)

	With diabetes; <55 years-old and not of black ethnicity; with CKD			Black ethnicity or >55 years-old		
**Antihypertensive regimen prior to optimisation**	**n**	**BP not on target, n (%)**	**Additional antihypertensive treatment[1]**	**n**	**BP not on target, n (%)**	**Additional antihypertensive treatment[1]**
ACEi/ARB+CCB+TD	2681	637 (23.8%)	None	1537	482 (31.4%)	None
ACEi/ARB+CCB+TD+Others	2173	643 (29.6%)	None	767	280 (36.5%)	None
**Subtotal**	**4854**	**1280 (26.4%)**		**2304**	**762 (33.1%)**	
ACEi/ARB only	9737	2333 (24%)	TD	4019	1115 (27.7%)	CCB
ACEi/ARB+CCB	8963	2155 (24%)	TD	5022	1571 (31.3%)	TD
ACEi/ARB+CCB+Others	5476	1358 (24.8%)	TD	1962	595 (30.3%)	TD
ACEi/ARB+Others	4865	983 (20.2%)	TD	2155	539 (25%)	CCB
ACEi/ARB+TD	1813	340 (18.8%)	CCB	1442	355 (24.6%)	CCB
ACEi/ARB+TD+Others	1179	281 (23.8%)	CCB	607	175 (28.8%)	CCB
CCB only	5345	1342 (25.1%)	ACEi/ARB	8574	2535 (29.6%)	TD
CCB+Others	2055	489 (23.8%)	ACEi/ARB	1320	347 (26.3%)	TD
CCB+TD	843	182 (21.6%)	ACEi/ARB	1535	419 (27.3%)	ACEi/ARB
CCB+TD+Others	443	99 (22.3%)	ACEi/ARB	347	97 (28%)	ACEi/ARB
Others only	1968	368 (18.7%)	ACEi/ARB	1064	238 (22.4%)	CCB
TD only	413	88 (21.3%)	ACEi/ARB	836	204 (24.4%)	CCB
TD+Others	304	63 (20.7%)	ACEi/ARB	271	64 (23.6%)	CCB
**Subtotal**	**43404**	**10081 (23.2%)**		**29154**	**8254 (28.3%)**	
No treatment	6073	1819 (30%)	ACEi/ARB	6039	2037 (33.7%)	CCB
**Total**	**54331**	**13180**		**37497**	**11053**	
**Total BP not on target with suboptimal treatment[2]**			**18335**			
**Total BP not on target with no treatment[3]**			**3856**			
**Total for antihypertensive treatment optimisation**			**22191**			

CKD, chronic kidney disease; BP, blood pressure; ACEi/ARB, angiotensin converting enzyme inhibitors or angiotensin receptor blockers; CCB, calcium channel blockers; TD, thiazide diuretics.
Others: beta blockers, spironolactone/K sparing, centrally acting antihypertensive agents, alpha blockers, or loop diuretics;
Target: systolic/diastolic BP <140/90 for <80 years old, or <150/90 for 80+ year old.
[1]Patients with BP not on target and on the third line treatment, i.e. ACEi/ARB+CCB+TD or ACEi/ARB+CCB+TD+Others were assumed optimally managed.
[2]Includes 18282 patients with known BP and 53 patients with unknown BP before imputation.
[3]Includes 3672 patients with known BP and 184 patients with unknown BP before imputation.

Wu R, *et al. BMJ Open* 2021; 11:e052884. doi: 10.1136/bmjopen-2021-052884

Table S12: Characteristics of patients with CVD or hypertension not optimally treated in the three east London CCGs

	Patients with Hypertension not optimally treated			Patients with CVD not optimally treated		
	Total	Antihypertensive treatment		Total	Cholesterol-lowering treatment	
		Suboptimal	Not treated		Suboptimal	Not treated
**N (%)**	**21954**	**18,282 (83%)**	**3672 (17%)**	**9062**	**5729 (63%)**	**3333 (37%)**
CCGs						
City and Hackney	6251 (23%)	4991 (80%)	1260 (20%)	2590 (36%)	1431 (55%)	1159 (45%)
Newham	10820 (28%)	9209 (85%)	1611 (15%)	4280 (45%)	2911 (68%)	1369 (32%)
Tower Hamlets	4883 (21%)	4082 (84%)	801 (16%)	2192 (31%)	1387 (63%)	805 (37%)
Age	59.1 (13.1)	60.1 (12.8)	54.3 (13.3)	69.3 (14.0)	71 (12.0)	66.3 (16.4)
Sex						
Female	10827 (24%)	9185 (85%)	1642 (15%)	3925 (44%)	2442 (62%)	1483 (38%)
Male	11127 (26%)	9097 (82%)	2030 (18%)	5137 (35%)	3287 (64%)	1850 (36%)
Ethnicity						
White	7179 (24%)	5888 (82%)	1291 (18%)	4115 (40%)	2462 (60%)	1653 (40%)
Black	6810 (28%)	5555 (82%)	1255 (18%)	1549 (43%)	875 (56%)	674 (44%)
South Asian	6315 (23%)	5518 (87%)	797 (13%)	2717 (33%)	2030 (75%)	687 (25%)
Other	1019 (21%)	852 (83.6%)	167 (16.4%)	453 (42%)	271 (60%)	182 (40%)
Not available	631 (30%)	469 (74.3%)	162 (25.7%)	228 (42%)	91 (40%)	137 (60%)
IMD						
Quintile 1 (least deprived)	118 (20%)	88 (74.58%)	30 (25.42%)	67 (39%)	42 (63%)	25 (37%)
Q2	269 (23%)	216 (80.3%)	53 (19.7%)	137 (43%)	77 (56%)	60 (44%)
Q3	979 (24%)	781 (79.8%)	198 (20.2%)	436 (41%)	261 (60%)	175 (40%)
Q4	9013 (25%)	7552 (84%)	1461 (16%)	3799 (41%)	2464 (65%)	1335 (35%)
Quintile 5 (most deprived)	11566 (24%)	9640 (83%)	1926 (17%)	4616 (36%)	2881 (62%)	1735 (38%)
Not available	9 (23%)	5 (55.56%)	4 (44.44%)	7 (78%)	4 (57%)	3 (43%)
Smoking status						
Non-smoker	14037 (25%)	11749 (84%)	2288 (16%)	4789 (42%)	3074 (64%)	1715 (36%)
Ex-smoker	4355 (22%)	3692 (85%)	663 (15%)	2628 (35%)	1717 (65%)	911 (35%)
Current smoker	3412 (28%)	2734 (80%)	678 (20%)	1607 (35%)	923 (57%)	684 (43%)
Not available	150 (39%)	107 (71.33%)	43 (28.67%)	38 (54.29%)	15 (39%)	23 (61%)
Diseases status						
Myocardial infarction	668 (18%)	639 (96%)	29 (4%)	1545 (25%)	1011 (65%)	534 (35%)
Angina	604 (18%)	573 (95%)	31 (5%)	1744 (37%)	1248 (72%)	496 (28%)
Other IHD	1699 (18%)	1615 (95%)	84 (5%)	4922 (34%)	3446 (70%)	1476 (30%)

Wu R, *et al. BMJ Open* 2021; 11:e052884. doi: 10.1136/bmjopen-2021-052884

PAD	347 (19%)	326 (94%)	21 (6%)	1101 (42%)	707 (64%)	394 (36%)
Stroke	1019 (19%)	932 (91%)	87 (9%)	3441 (44%)	1931 (56%)	1510 (44%)
Atrial fibrillation	684 (17%)	657 (96%)	27 (4%)	1044 (42%)	638 (61%)	406 (39%)
Heart failure	671 (18%)	649 (97%)	22 (3%)	1041 (34%)	680 (65%)	361 (35%)
Diabetes	5949 (18%)	5524 (93%)	425 (7%)	3267 (32%)	2582 (79%)	685 (21%)
Chronic kidney disease	3058 (19%)	2846 (93%)	212 (7%)	2618 (42%)	1853 (71%)	765 (29%)
Hypertension				5934 (38%)	4134 (70%)	1800 (30%)
Clinical measures						
Total cholesterol (mmol/L)	4.7 (1.2)	4.6 (1.2)	5.1 (1.1)	4.3 (1.1)	4 (1.0)	4.9 (1.1)
Triglycerides (mmol/L)	1.5 (1.1)	1.5 (1.11)	1.5 (1.07)	1.5 (0.94)	1.5 (0.89)	1.5 (1.03)
HDL (mmol/L)	1.4 (0.39)	1.4 (0.39)	1.5 (0.41)	1.4 (0.38)	1.4 (0.36)	1.4 (0.41)
Creatinine (µmol/L)	90 (66)	91 (68)	85 (51)	99 (71)	100 (69)	96 (74)
Systolic blood pressure (mmHg)	147 (13)	147 (13)	148 (14)	130 (15)	129 (15)	130 (16)
Diastolic blood pressure (mmHg)	87 (11)	86 (11)	91 (11)	75 (10)	74 (10)	76 (10)

Column %s presented for Totals and row %s for subgroups on optimal and non-optimal treatment.

CVD, cardiovascular disease; CCG, clinical commissioning group; IMD, index of multiple deprivation; IHD, ischaemic heart disease; PAD, peripheral artery disease; HDL, high-density lipoprotein.

Supplementary Table S13: Predicted vascular events avoided of fully optimised antihypertensive and statin treatment

Age (years)	Patients with hypertension not on optimal antihypertensive treatment N = 22191	Patients with prior CVD not on optimal statin treatment N = 9062
**Non-fatal MVEs avoided per optimised patient (95% CI)**		
<50	0.24 (0.12 to 0.33)	0.34 (0.25 to 0.42)
50-59	0.17 (0.08 to 0.24)	0.23 (0.17 to 0.29)
60-69	0.13 (0.05 to 0.20)	0.15 (0.11 to 0.19)
70-79	0.08 (0.02 to 0.13)	0.09 (0.06 to 0.11)
⩾80	0.05 (0.00 to 0.09)	0.05 (0.04 to 0.07)
**Non-fatal OVEs avoided per optimised patient (95% CI)**		
<50	0.30 (0.20 to 0.38)	0.16 (0.02 to 0.30)
50-59	0.22 (0.15 to 0.29)	0.11 (0.01 to 0.21)
60-69	0.18 (0.12 to 0.24)	0.07 (0.00 to 0.14)
70-79	0.14 (0.09 to 0.19)	0.04 (0.00 to 0.08)
⩾80	0.10 (0.06 to 0.14)	0.02 (0.00 to 0.05)
**Vascular deaths avoided per optimised patient (95% CI)**		
<50	0.07 (0.03 to 0.11)	0.05 (0.04 to 0.06)
50-59	0.07 (0.03 to 0.10)	0.04 (0.03 to 0.05)
60-69	0.07 (0.03 to 0.10)	0.03 (0.02 to 0.04)
70-79	0.06 (0.02 to 0.09)	0.02 (0.02 to 0.03)
⩾80	0.06 (0.02 to 0.09)	0.02 (0.02 to 0.03)

CVD, cardiovascular disease; MVE, major vascular event; OVE, other vascular event.
1203 patients with hypertension and previous CVD included in both patient categories.

Wu R, *et al. BMJ Open* 2021; 11:e052884. doi: 10.1136/bmjopen-2021-052884

Table S14 Predicted total lifetime health gains, hospital cost savings and extra medication costs with optimised antihypertensive and statin treatment in target populations in east London

	Patients with hypertension not on optimal antihypertensive treatment N = 22,191	Patients with prior CVD not on optimal statin treatment N = 9062
Life years gained	22228 (9234 to 33296)	4034 (2948 to 5059)
QALYs gained	16698 (9485 to 22482)	2616 (2022 to 3184)
Non-fatal MVEs avoided	3362 (1592 to 4949)	1255 (905 to 1571)
Non-fatal OVEs avoided	4630 (3102 to 6070)	580 (15 to 1124)
Vascular deaths avoided	1450 (602 to 2172)	277 (202 to 345)
Hospital cost savings (UK£)	41,069,942 (6,894,396 to 74,751,579)	11,603,287 (7,243,739 to 15,597,438)
Extra medication costs (UK£)	10,434,241 (10,139,402 to 10,685,867)	2,073,070 (2,020,288 to 2,123,300)

CVD, cardiovascular disease; QALY, quality-adjusted life year; MVE, major vascular event; OVE, other vascular event.

Wu R, *et al. BMJ Open* 2021; 11:e052884. doi: 10.1136/bmjopen-2021-052884

Table S15: Predicted lifetime gains in survival and QALYs, hospital care cost saved, and additional medication cost of fully optimised statin and antihypertensive treatments, separately for previously sub-optimally treated and not treated

Age at start (years)	Patients with hypertension not on optimal antihypertensive treatment		Patients with CVD not on optimal cholesterol-lowering treatment	
	Suboptimal to optimal, N = 18335	No treatment to optimal, N = 3856	Suboptimal to optimal, N = 5729	No treatment to optimal, N = 3333
	Life years gained (95% CI), undiscounted			
<50	1.49 (0.62 to 2.24)	0.97 (0.40 to 1.49)	0.47 (0.34 to 0.61)	1.27 (0.92 to 1.64)
50-59	1.15 (0.47 to 1.72)	0.79 (0.33 to 1.2)	0.40 (0.29 to 0.52)	1.10 (0.80 to 1.39)
60-69	0.95 (0.39 to 1.42)	0.66 (0.28 to 0.99)	0.29 (0.21 to 0.37)	0.85 (0.63 to 1.07)
70-79	0.68 (0.28 to 1.01)	0.48 (0.20 to 0.72)	0.18 (0.13 to 0.23)	0.61 (0.45 to 0.76)
≥80	0.56 (0.23 to 0.83)	0.38 (0.16 to 0.57)	0.11 (0.08 to 0.13)	0.41 (0.30 to 0.51)
	Life years gained (95% CI), discounted at 1.5% per annum			
<50	0.86 (0.36 to 1.28)	0.54 (0.22 to 0.83)	0.28 (0.2 to 0.37)	0.72 (0.52 to 0.93)
50-59	0.74 (0.31 to 1.11)	0.51 (0.21 to 0.77)	0.27 (0.19 to 0.34)	0.73 (0.53 to 0.92)
60-69	0.67 (0.28 to 1.01)	0.46 (0.19 to 0.7)	0.21 (0.15 to 0.27)	0.61 (0.45 to 0.77)
70-79	0.52 (0.21 to 0.77)	0.37 (0.15 to 0.55)	0.14 (0.1 to 0.18)	0.48 (0.35 to 0.59)
≥80	0.46 (0.19 to 0.69)	0.31 (0.13 to 0.47)	0.09 (0.06 to 0.11)	0.35 (0.25 to 0.43)
	QALYs gained (95% CI), undiscounted			
<50	1.19 (0.69 to 1.61)	0.87 (0.51 to 1.19)	0.32 (0.24 to 0.40)	0.90 (0.69 to 1.12)
50-59	0.87 (0.50 to 1.17)	0.68 (0.39 to 0.92)	0.27 (0.21 to 0.33)	0.75 (0.58 to 0.92)
60-69	0.66 (0.37 to 0.90)	0.53 (0.30 to 0.73)	0.18 (0.14 to 0.22)	0.57 (0.44 to 0.68)
70-79	0.44 (0.24 to 0.59)	0.36 (0.20 to 0.49)	0.11 (0.09 to 0.13)	0.38 (0.30 to 0.46)
≥80	0.32 (0.17 to 0.44)	0.25 (0.13 to 0.34)	0.06 (0.04 to 0.07)	0.23 (0.18 to 0.28)
	QALYs gained (95% CI), discounted at 1.5% per annum			
<50	0.73 (0.45 to 0.98)	0.52 (0.31 to 0.7)	0.2 (0.15 to 0.25)	0.54 (0.41 to 0.66)
50-59	0.59 (0.35 to 0.79)	0.46 (0.27 to 0.62)	0.18 (0.14 to 0.23)	0.52 (0.4 to 0.63)
60-69	0.49 (0.28 to 0.66)	0.39 (0.22 to 0.53)	0.14 (0.11 to 0.17)	0.42 (0.33 to 0.5)
70-79	0.34 (0.19 to 0.47)	0.28 (0.16 to 0.38)	0.09 (0.07 to 0.11)	0.3 (0.24 to 0.36)
≥80	0.27 (0.14 to 0.37)	0.2 (0.11 to 0.29)	0.05 (0.04 to 0.06)	0.19 (0.15 to 0.23)
	Hospital care cost saved (UK£) (95% CI), undiscounted			
<50	3313 (1059 to 5507)	2512 (931 to 4032)	1533 (1012 to 2015)	4383 (3053 to 5589)
50-59	2141 (412 to 3835)	1716 (453 to 2912)	1264 (814 to 1684)	3498 (2326 to 4544)
60-69	1475 (6 to 2960)	1259 (193 to 2296)	825 (490 to 1145)	2570 (1655 to 3396)
70-79	837 (-245 to 1940)	777 (-53 to 1591)	450 (233 to 664)	1586 (908 to 2220)
≥80	348 (-585 to 1271)	389 (-292 to 1061)	183 (53 to 308)	760 (299 to 1212)
	Hospital care cost saved (UK£) (95% CI), discounted at 1.5% per annum			
<50	2716 (1298 to 4037)	1975 (990 to 2875)	1235 (887 to 1556)	3404 (2556 to 4159)
50-59	1910 (724 to 3070)	1491 (621 to 2330)	1082 (763 to 1380)	2985 (2157 to 3718)
60-69	1411 (313 to 2492)	1159 (379 to 1923)	747 (492 to 989)	2304 (1624 to 2926)
70-79	852 (-3 to 1727)	758 (107 to 1403)	428 (250 to 600)	1501 (952 to 2028)
≥80	399 (-392 to 1172)	406 (-169 to 973)	183 (68 to 291)	755 (350 to 1148)
	Additional medication costs (UK£) (95% CI), undiscounted			
<50	794 (771 to 812)	486 (475 to 495)	353 (345 to 361)	894 (874 to 914)
50-59	570 (554 to 583)	321 (314 to 328)	240 (233 to 245)	608 (593 to 623)
60-69	412 (399 to 423)	231 (225 to 236)	158 (153 to 162)	444 (433 to 455)
70-79	278 (269 to 286)	161 (157 to 165)	67 (65 to 69)	242 (235 to 249)
≥80	165 (158 to 172)	105 (102 to 108)	17 (17 to 18)	119 (114 to 124)
	Additional medication costs (UK£) (95% CI), discounted at 1.5% per annum			

<50	584 (571 to 594)	352 (346 to 357)	266 (261 to 270)	653 (642 to 664)
50-59	448 (438 to 457)	253 (248 to 256)	191 (187 to 195)	484 (474 to 493)
60-69	341 (332 to 349)	190 (187 to 194)	132 (129 to 135)	371 (363 to 379)
70-79	241 (234 to 247)	140 (136 to 142)	59 (57 to 60)	212 (206 to 217)
≥80	149 (143 to 154)	95 (92 to 97)	16 (15 to 17)	108 (104 to 112)

CVD, cardiovascular disease; QALY, quality-adjusted life year.

Wu R, *et al. BMJ Open* 2021; 11:e052884. doi: 10.1136/bmjopen-2021-052884

Supplementary Table S16: Optimising antihypertensive and statin treatments in patients with both hypertension and CVD and not on optimal antihypertensive nor cholesterol-lowering treatment (N=1203)

Age (years)	Number patients	Life years gained per patient (95% CI)	QALYs gained per patient (95% CI)	Hospital care cost savings (UK£) per patient (95% CI)	Additional medication costs (UK£) per patient (95% CI)
<50	58	2.53 (1.37 to 3.53)	1.66 (1 to 2.22)	5284 (2482 to 7943)	1181 (1138 to 1217)
50-59	210	2.02 (1.13 to 2.78)	1.29 (0.8 to 1.7)	4433 (2099 to 6735)	861 (827 to 888)
60-69	344	1.57 (0.84 to 2.18)	0.93 (0.57 to 1.23)	3072 (1134 to 5066)	621 (595 to 641)
70-79	365	1.08 (0.57 to 1.51)	0.61 (0.37 to 0.81)	1741 (290 to 3253)	369 (353 to 382)
≥80	226	0.84 (0.44 to 1.18)	0.44 (0.26 to 0.59)	710 (-464 to 1908)	204 (192 to 214)

CVD, cardiovascular disease; QALY, quality-adjusted life year.

Figure S1: Projected reductions in non-fatal major vascular events and vascular deaths, and extra life years and QALYs with optimised antihypertensive and statin treatment over 5, 10 years and lifetime: scenarios with optimising extra 10%, 20% and all patients not on optimal treatment

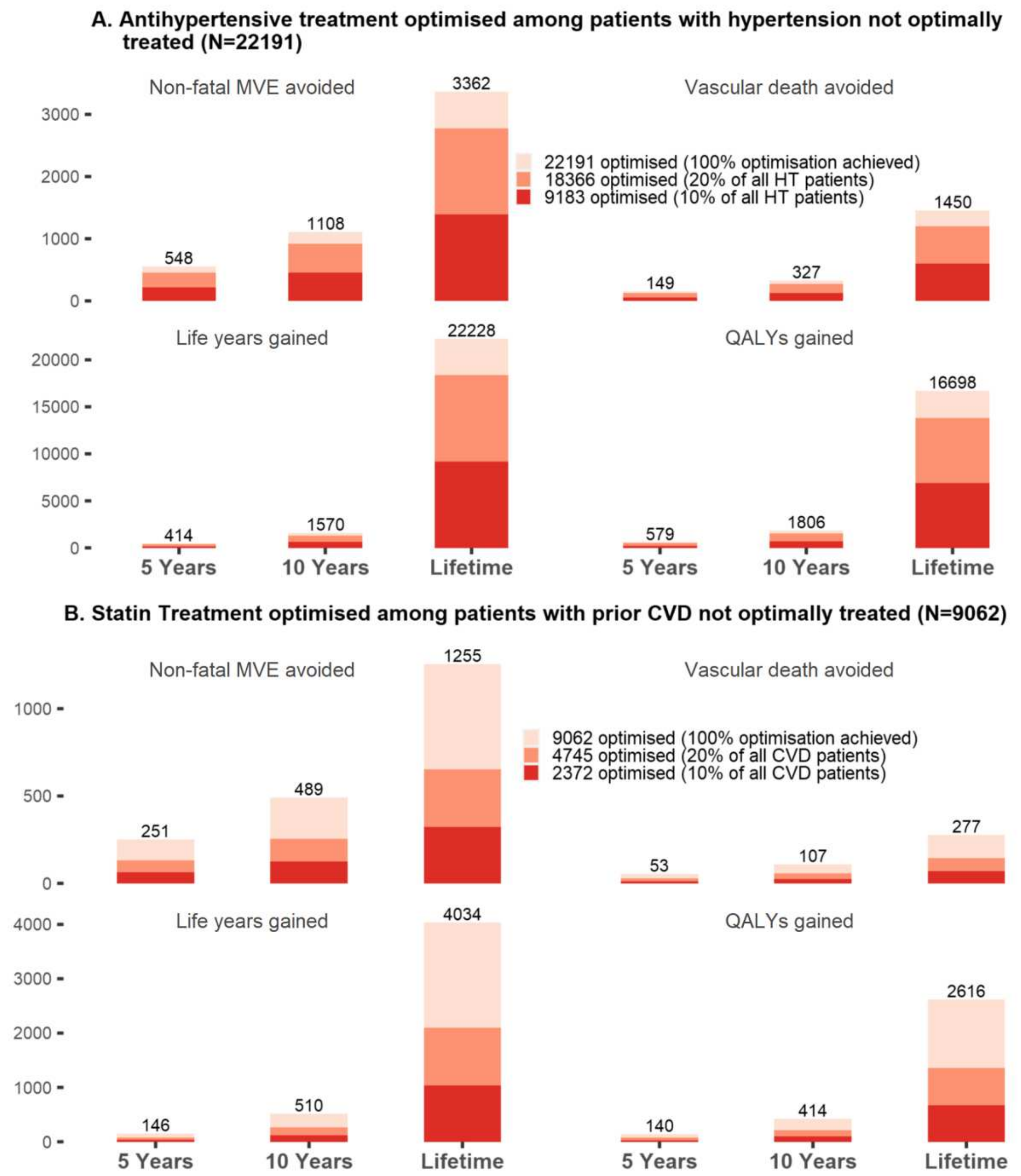

CVD, cardiovascular disease; HT, hypertension; MVE, major vascular event; QALY, quality-adjusted life year.

References

1. Office for National Statistics. Population estimates - local authority based by single year of age 2019 [Available from: https://www.nomisweb.co.uk/datasets/pestsyoala accessed 2/12/2020.
2. Office for National Statistics. Mortality statistics - underlying cause, sex and age 2019 [Available from: https://www.ons.gov.uk/peoplepopulationandcommunity/birthsdeathsandmarriages/deaths/bulletins/deathsregistrationsummarytables/2019 accessed 2/12/2020.
3. Hippisley-Cox J, Coupland C, Brindle P. Development and validation of QRISK3 risk prediction algorithms to estimate future risk of cardiovascular disease: prospective cohort study. *BMJ* 2017;357:j2099.
4. British Heart Foundation. Heart and Circulatory Disease Statistics 2020 [Available from: https://www.bhf.org.uk/what-we-do/our-research/heart-statistics/heart-statistics-publications/cardiovascular-disease-statistics-2020 accessed 29/1/2021.
5. Blood Pressure Lowering Treatment Trialists' Collaboration. Effects of different blood-pressure-lowering regimens on major cardiovascular events: results of prospectively-designed overviews of randomised trials. *Lancet* 2003;362(9395):1527-35. doi: https://doi.org/10.1016/S0140-6736(03)14739-3
6. Blood Pressure Lowering Treatment Trialists’ Collaboration. Blood pressure lowering and major cardiovascular events in people with and without chronic kidney disease: meta-analysis of randomised controlled trials. *BMJ* 2013;347:f5680. doi: 10.1136/bmj.f5680
7. Cholesterol Treatment Trialists’ (CTT) Collaboration. Efficacy and safety of more intensive lowering of LDL cholesterol: a meta-analysis of data from 170 000 participants in 26 randomised trials. *Lancet* 2010;376(9753):1670-81.
8. NICE. Lipid modification: cardiovascular risk assessment and the modification of blood lipids for the primary and secondary prevention of cardiovascular disease. London: National Institute for Health and Care Excellence, 2014.
9. Knopp R, Gitter H, Truitt T, et al. Effects of ezetimibe, a new cholesterol absorption inhibitor, on plasma lipids in patients with primary hypercholesterolemia. *Eur Heart J* 2003;24(8):729-41.
10. Ballantyne CM, Abate N, Yuan Z, et al. Dose-comparison study of the combination of ezetimibe and simvastatin (Vytorin) versus atorvastatin in patients with hypercholesterolemia: the Vytorin Versus Atorvastatin (VYVA) study. *Am Heart J* 2005;149(3):464-73.
11. Ballantyne CM, Weiss R, Moccetti T, et al. Efficacy and safety of rosuvastatin 40 mg alone or in combination with ezetimibe in patients at high risk of cardiovascular disease (results from the EXPLORER study). *American Journal of Cardiology* 2007;99(5):673-80.
12. Melani L, Mills R, Hassman D, et al. Efficacy and safety of ezetimibe coadministered with pravastatin in patients with primary hypercholesterolemia: a prospective, randomized, double-blind trial. *Eur Heart J* 2003;24(8):717-28.
13. Stojakovic T, De Campo A, Scharnagl H, et al. Differential effects of fluvastatin alone or in combination with ezetimibe on lipoprotein subfractions in patients at high risk of coronary events. *Eur J Clin Invest* 2010;40(3):187-94.
14. Ballantyne CM, Hoogeveen RC, Raya JL, et al. Efficacy, safety and effect on biomarkers related to cholesterol and lipoprotein metabolism of rosuvastatin 10 or 20 mg plus ezetimibe 10 mg vs. simvastatin 40 or 80 mg plus ezetimibe 10 mg in high-risk patients: results of the GRAVITY randomized study. *Atherosclerosis* 2014;232(1):86-93.
15. Law MR, Wald NJ, Rudnicka A. Quantifying effect of statins on low density lipoprotein cholesterol, ischaemic heart disease, and stroke: systematic review and meta-analysis. *BMJ* 2003;326(7404):1423.
16. Curtis L, Burns A. Unit Costs of Health and Social Care 2019. Canterbury: Personal Social Services Research Unit, University of Kent, 2019.

17. NHS Business Services Authority. Prescription Cost Analysis - England 2019. Newcastle upon Tyne, 2020.
